# Assessing the Effect of Breast Density on Screening Performance in a Population‐Based Program

**DOI:** 10.1155/tbj/9750357

**Published:** 2026-09-03

**Authors:** Chirag Mistry, Richard Walton, Matthew Warner-Smith, Kan Ren, David Roder, Bronwyn Morley, Tracey O’Brien

**Affiliations:** ^1^ Cancer Institute NSW, Sydney, New South Wales, Australia; ^2^ Macquarie University, Sydney, New South Wales, Australia, mq.edu.au; ^3^ Cancer Epidemiology & Population Health, Adelaide University, Adelaide, South Australia, Australia, adelaide.edu.au

**Keywords:** breast cancer, breast density, cancer incidence, diagnostic performance, interval cancers, population-based screening, screening mammography

## Abstract

**Background:**

Breast density is both an independent risk factor for breast cancer and a major determinant of mammographic diagnostic performance. However, its impact on overall screening performance within population‐based screening programs is less well understood.

**Methods:**

This retrospective cohort study included women aged 50–74 years screened through BreastScreen NSW between 1 January 2016 and 31 December 2017, excluding first‐time screeners above 70. Breast density was assessed using Lunit INSIGHT MMG (v1.1.7) generating an ordinal density score (1–10) and corresponding BI‐RADS category (A–D). Screen‐detected and interval cancers were ascertained via the NSW BreastScreen Information System and the NSW Cancer Registry. Standard screening performance metrics were calculated stratified by age, breast density and screening round.

**Results:**

The cohort comprised 564,681 screening episodes, with 4758 cancers diagnosed (3756 screen‐detected; 1002 interval). Cancer incidence rates and screening performance varied by breast density. The proportion of cancers that were interval cancers increased markedly with increasing density, from 10.9% of cancers in category A (least dense) to 46.6% in category D. Recall rates followed an N‐shaped pattern, peaking in density category C, while specificity mirrored this relationship.

**Conclusion:**

The effectiveness of conventional two‐dimensional mammography is strongly dependent on breast density, with reduced sensitivity and increased interval cancer rates for clients with extremely dense breasts. By utilising a more granular measure of breast density in a large population screening cohort, this study identified nonlinear relationships between breast density and screening performance metrics, providing evidence to support future evaluation of more individualised screening approaches.

**Highlights**
• Machine‐reading enables scalable breast density assessment in screening programs.
• Screening performance was shown to vary substantially across breast density groups.
• Higher breast density associated with lower sensitivity and more interval cancers.
• Recall rates and specificity show N‐shaped relationships across density categories.
• Findings provide evidence of inequities and support risk‐stratified screening.

## 1. Introduction

Breast cancer is the most commonly diagnosed cancer among women in Australia and remains a leading cause of cancer‐related mortality in the country. Each year, approximately 20,000 women are diagnosed with the illness, accounting for over a quarter of all female cancer diagnoses [1]. It is estimated that the risk of being diagnosed with breast cancer over a lifetime for women is now 1 in 7 [2]. Despite this, advancements in early detection and treatment have meant that survival rates are observed to have improved considerably in recent years, with 5‐year survival rising from 75% (1987–1991) to 93% (2017–2021) [1].

Population‐based screening programs have been instrumental in allowing the systematic early detection of instances of breast cancer and improving the long‐term outcomes of those diagnosed [3, 4]. For instance, in New South Wales, the BreastScreen NSW (BSNSW) program screens over 350,000 women each year and detected over 2500 cancers in 2024 (BSNSW internal data, 2024). The program invites women aged 50–74 years to attend biennial screening, with women aged 40 and over also eligible to participate. Although screening programs have demonstrated clear population‐level benefits, they are largely based on a uniform screening model despite substantial variation in breast cancer risk and mammographic detectability between individuals in a population. Breast cancer risk is influenced by many factors such as age, family history, genetic susceptibility, breast density, obesity and alcohol use [5, 6]. Among these, breast density is of particular interest due to its role both as an independent risk factor for breast cancer and as a notable limitation in mammographic diagnostic accuracy.

Many studies have shown that women with dense breast tissue have a higher likelihood of developing breast cancer compared to those with less dense tissue [7–9]. In a recent meta‐analysis, Bodewes et al. reported that having BI‐RADS density D (extremely dense breast tissue) resulted in a 3.89‐fold increase in breast cancer risk compared to density A (almost entirely fatty tissue) [10]. Some studies report this number to be higher [7, 11]. In addition, increased breast density reduces the sensitivity of screening mammography as potential cancers can be obscured by dense breast tissue (known as ‘masking’) [12]. Freer showed that mammographic sensitivity can drop from 86%–89% to 62%–68% for women with extremely dense breasts, leading to delayed diagnoses and poorer outcomes [13]. Comparable large cohort studies in Spain and the United States have produced strikingly similar findings [14, 15].

Recent years have seen the emergence of commercially available deep learning–based machine reading algorithms capable of interpreting digital mammographic images and evaluating breast density. One such product, Lunit’s INSIGHT MMG, can categorise mammographic images into an approximation of the BI‐RADS classification system (A–D, with A = almost entirely fatty, D = extremely dense) at scale. In a recent study, Walton et al. demonstrated ‘substantial’ inter‐reader reliability between the breast density assessment of INSIGHT MMG and program radiologists in a sample of 15,518 individual clients (weighted kappa coefficient of 0.72 (95% CI 0.71–0.73) [16], supporting its suitability for use in population‐based screening programs.

Attention on the assessment of breast density has become increasingly focused following BreastScreen Australia’s recommendation that women should be informed of their breast density as measured on their screening mammogram [17], and may seek advice from their general practitioner (GP) or breast cancer specialist to understand its implications. As breast density information becomes routinely available to screening participants, screening programs are likely to face increased scrutiny on how that information can be used to better serve their clients. A recent study [18] found that women notified of their dense breasts reported feeling significantly more anxious and confused, and rather than feel more informed to make decisions about their breast health, wanted to be guided by their GPs. Consequently, there is a need to better understand breast density as a risk factor and its influence on the performance of population‐based screening programs. Although previous studies have demonstrated associations between breast density and breast cancer risk, few have evaluated screening performance across an entire population‐based screening cohort, including linking to complete screening outcomes. This study aims to extend the existing evidence by characterising the distribution of breast density across the New South Wales screening population and examining how key screening performance metrics, including interval cancer outcomes, vary across breast density groups.

## 2. Methods

### 2.1. Study Design and Study Population

This retrospective population‐based cohort study included all clients who screened with the BSNSW program between 1 January 2016 and 31 December 2017. Detailed methods have previously been described in Warner‐Smith et al. [19]. The 2‐year study period coincides with a complete screening cycle for the program, capturing the routine BSNSW screening population over its biennial screening period.

For clients who had more than one screening episode during the study period, only the first screening episode was retained. Clients outside the target range of the program (below 50 years or aged 75 and above) were excluded from the study sample. Additionally, initial screeners aged over 70 years were also removed, as this cohort is unusual for joining the screening program so late and has a much higher incidence rate of cancer. Screening episodes resulting in technical recalls were also removed. The final analysis dataset included screening episode details and associated reading outcomes.

### 2.2. Breast Density Assessment and Cancer Outcome

The study cohort was enriched with a measurement of breast density from the reading software (Lunit INSIGHT MMG version 1.1.7) for each client at the time of their screening episode. Since density varies across breast tissue, the episode‐level classification was based on the maximum density across all mammographic images. The machine reader provided an ordinal breast density score from 1 to 10, which was converted to an approximate BI‐RADS category using predefined cut‐offs recommended by Lunit.

Cancers diagnosed through the program during a client’s screening episode, as well as cancers diagnosed outside the program within 24 months of the screening episode (interval cancers), were identified from the BSNSW’s BreastScreen Information System (BIS) and the NSW Cancer Registry. These diagnoses were treated as the ground truth for the calculation of standard screening performance metrics. The incidence rate of all cancer cases and of interval cancer cases was also computed.

### 2.3. Outcome Measures

Screen reading performance metrics (sensitivity, specificity, positive predictive value and recall rate) were compared with the final clinical outcome recorded in the NSW Cancer Registry. Estimates of cancer incidence rates (all, screen‐detected and interval cancers) were based on the episode‐level outcome of the screening episode (i.e., following further diagnostics after abnormalities were identified at reading). Results were stratified by age and density. Breast density was summarised using BI‐RADS categories to facilitate comparison with existing literature, while figures present the ordinal breast density score (1–10) to more clearly represent the underlying distribution.

### 2.4. Statistical Analysis

Differences in cancer incidence across groups were assessed using two‐sample *Z* tests for proportions. All analyses were undertaken using SAS EG 8.2 and R version 4.4.2. Generalised additive models (GAMs) with cubic regression splines were fitted using the mgcv package in R to explore nonlinear relationships between breast density and screening performance metrics. Observations were weighted by subgroup size when fitting smoothed curves. Data cleaning and manipulation were undertaken using the dplyr, tidyr and stringr packages.

This study has ethical approval from the NSW Health Population Health Services Research Ethics Committee (2022/ETH02397).

## 3. Results

**FIGURE 1:**
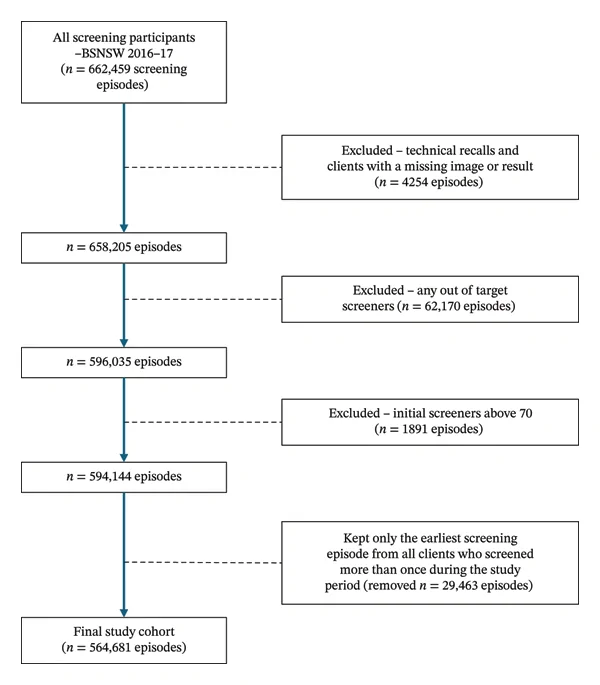
Flow diagram of clients for the derivation of the study cohort.

Between 1 January 2016 and 31 December 2017, there were 662,459 screening episodes recorded. Applying the exclusions (Figure 1) resulted in a study cohort comprising 564,681 screening exams of 564,681 clients, of whom 58,004 (10.3%) were initial screeners. The mean age of the cohort was 61.0 years. There were 4758 cases of cancer in the cohort, 3756 of which were screen‐detected with the remaining 1002 being interval cancers identified through linkage with the NSW Cancer Registry.

The majority of clients (64.0%) were classified as having breast density A (almost entirely fatty) or density B (scattered fibroglandular tissue). In contrast, only 2.9% of clients were classified as having density D (extremely dense breasts) (Table 1). Mean age for clients with breast density A was 62.5 (s.d. = 6.6), and this value decreased with increasing breast density—the youngest cohort was clients with breast density D with a mean age of 57.8 (s.d. = 6.7). The largest proportion of initial screeners was also from the cohort with breast density D, 20.4%, compared to only 8.2% of those with breast density A, consistent with initial screeners generally being younger than those who have screened with the program before.

**TABLE 1:** Population summary table.

Breast density	A	B	C	D
Classification	*N* = 99,658 (17.6%)	*N* = 261,975 (46.4%)	*N* = 186,894 (33.1%)	*N* = 16,154 (2.9%)
Mean age (SD)	62.5 (6.6)	61.8 (6.7)	59.7 (6.7)	57.9 (6.7)
Median age	63.0	62.0	59.0	56.0
25th percentile	57.0	56.0	54.0	52.0
75th percentile	68.0	67.0	65.0	62.0
IQR	11.0	11.0	11.0	10.0
Age groups				
50–59	34,463 (34.6%)	102,556 (39.1%)	98,070 (52.5%)	10,353 (64.1%)
60–69	47,550 (47.7%)	118,238 (45.1%)	69,822 (37.4%)	4574 (28.3%)
70–74	17,645 (17.7%)	41,181 (15.7%)	19,002 (10.2%)	1227 (7.6%)
Screening round				
Initial	8186 (8.2%)	25,038 (9.6%)	21,488 (14.2%)	3292 (20.4%)
Subsequent	91,472 (91.8%)	236,937 (90.4%)	160,406 (85.8%)	12,862 (79.6%)

**FIGURE 2:**
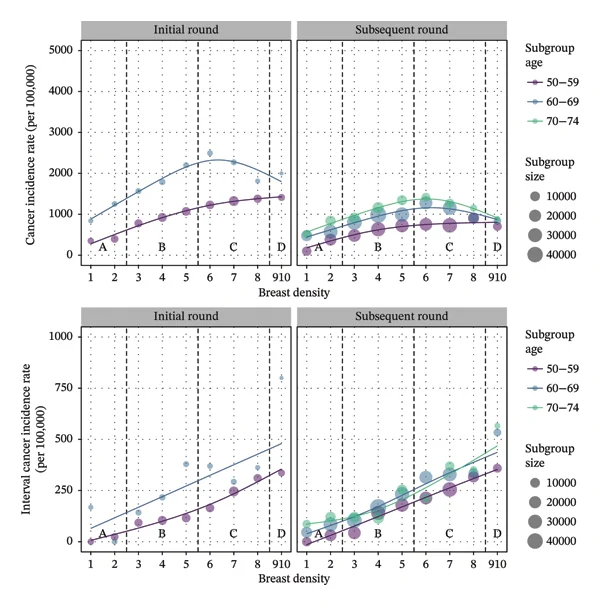
Incidence rate of all cancers and interval cancers by age group, screening round and breast density (*x* axis, ordinal scale 1–10).

The overall cancer incidence in the study cohort was 843 cases per 100,000 clients. Incidence was similar among clients with breast density B (853 per 100,000) and density D (891 per 100,000; *p* = 0.6) (Table 2). In contrast, incidence was substantially lower among clients with density A (484 per 100,000; *p* < 0.001) and significantly higher among those with density C (*p* < 0.001), when compared with density B, the largest density group in the cohort. These differences produced an N‐shaped relationship between breast density and cancer incidence, with a peak observed in density C (Figure 2 and Table 3).

**TABLE 2:** Cancer incidence rates and counts summary table.

Breast density classification	A	B	C	D	Total
	*N* = 482 (10.1%)	*N* = 2235 (47.0%)	*N* = 1897 (39.9%)	*N* = 144 (3.0%)	*N* = 4758
Cancer incidence rate (per 100,000)	484	853	1015	891	843
Initial	538	1090	1404	1428	1168
Subsequent	479	828	951	754	802
Age groups					
50–59	299	665	878	898	709
60–69	536	929	1149	875	914
70–74	703	1102	1231	896	1041
Cancer type					
Screen‐detected	427	710	750	472	666
Interval	57	144	267	421	177
Cancer counts by type (interval or screen‐detected)					
Screen‐detected	425 (88.2%)	1857 (83.1%)	1398 (73.7%)	76 (52.8%)	3756 (78.9%)
Age group					
50–59	97 (20.1%)	558 (25.0%)	628 (33.1%)	57 (39.6%)	1340 (28.2%)
60–69	222 (46.1%)	906 (40.5%)	590 (31.1%)	19 (13.2%)^∗^	2416 (50.8%)^∗^
70–74	106 (22.0%)	393 (17.6%)	180 (9.5%)		
Interval	57 (11.8%)	378 (16.9%)	499 (26.3%)	68 (47.2%)	1002 (21.1%)
Age group					
50–59	6 (1.2%)	124 (5.5%)	233 (12.3%)	36 (25.0%)	399 (8.4%)
60–69	33 (6.8%)	193 (8.6%)	212 (11.2%)	25 (17.4%)	463 (9.7%)
70–74	18 (3.7%)	61 (2.7%)	54 (2.8%)	7 (4.9%)	140 (2.9%)

^∗^The values represent the total for both the 60–69 and 70–74 age groups, and that they were combined to avoid reporting a count under 5.

**TABLE 3:** Screening performance metrics for different age groups and screening round.

Metric		Breast density							
		A		B		C		D	
Screening round	Age group	Initial	Subsequent	Initial	Subsequent	Initial	Subsequent	Initial	Subsequent
Population size	50–59	6358	28,105	20,343	82,213	23,420	74,650	3033	7320
	60–69	1828	45,722	4695	113,543	3068	66,754	259	4315
	70–74	NA	17,645	NA	41,181	NA	19,002	NA	1227

Cancer count (total)	50–59	24	79	188	494	303	558	42	51
	60–69	20	235	85	1014	69	733	5	35
	70–74	NA	124	NA	454	NA	234	NA	11

Cancer incidence rate per 100,000 (95% CI)	50–59	377 (242–561)	281 (223–350)	924 (797 – 1065)	601 (549–656)	1294 (1153 – 1447)	747 (687–812)	1385 (1000 – 1867)	697 (519–915)
	60–69	1094 (670 – 1685)	514 (450–584)	1810 (1449 – 2234)	893 (839–949)	2249 (1754 – 2838)	1098 (1020 – 1180)	1931 (630 – 4447)	811 (566 – 1126)
	70–74	NA	703 (585–837)	NA	1102 (1004–1208)	NA	1231 (1080 – 1399)	NA	896 (448 – 1598)

Interval cancer incidence rate per 10,000 (95% CI)	50–59	1.6 (0–8.8)	1.8 (0.6–4.2)	10.3 (6.4–15.8)	12.5 (10.2–15.2)	23.5 (17.7–30.6)	23.8 (20.5–27.6)	33 (15.8–60.6)	35.5 (23.2–52.0)
	60–69	5.5 (0.1–8.8)	7.0 (4.8–9.9)	23.4 (11.7–41.9)	16.0 (13.8–18.5)	32.6 (15.6–59.9)	30.3 (26.2–34.7)	77.2 (9.4–276.1)	53.3 (33.8–79.9)
	70–74	NA	10.2 (6.0–16.1)	NA	14.8 (11.3–19.0)	NA	28.4 (21.4–37.1)	NA	57.0 (23.0–117.2)

Recall rates (%) (95% CI)	50–59	6.6 (6.0–7.2)	1.8 (1.6–1.9)	11.1 (10.7–11.5)	3.1 (2.9–3.2)	11.7 (11.3–12.1)	3.6 (3.5–3.8)	8.5 (7.5–9.6)	3.2 (2.8–3.6)
	60–69	6.0 (5.0–7.2)	2.2 (2.0–2.3)	11.4 (10.5–12.4)	3.2 (3.1–3.3)	11.1 (10.0–12.2)	3.4 (3.2–3.5)	8.5 (5.4–12.6)	2.1 (1.7–2.6)
	70–74	NA	2.4 (2.2–2.7)	NA	3.4 (3.2–3.6)	NA	3.7 (3.5–4.0)	NA	2.7 (1.9–3.8)

False positive rates (%) (95% CI)	50–59	6.2 (5.6–6.8)	1.5 (1.3–1.6)	10.3 (9.9–10.7)	2.6 (2.5–2.7)	10.6 (10.2–11.0)	3.1 (3.0–3.2)	7.5 (6.5–8.4)	2.8 (2.4–3.2)
	60–69	5.0 (4.0–6.1)	1.7 (1.6–1.8)	9.8 (9.0–10.7)	2.5 (2.4–2.5)	9.1 (8.1–10.2)	2.6 (2.4–2.7)	7.3 (4.5–11.2)	1.8 (1.4–2.3)
	70–74	NA	1.8 (1.4–2.3)	NA	2.4 (2.3–2.6)	NA	2.8 (2.5–3.0)	NA	2.3 (1.5–3.3)

False negative rates (%) (95% CI)	50–59	0.02 (0.00–0.09)	0.02 (0.01–0.04)	0.10 (0.06–0.15)	0.11 (0.09–0.14)	0.20 (0.15–0.27)	0.22 (0.19–0.26)	0.33 (0.16–0.61)	0.33 (0.21–0.49)
	60–69	0.05 (0.00–0.30)	0.06 (0.04–0.08)	0.19 (0.09–0.36)	0.15 (0.13–0.17)	0.29 (0.13–0.56)	0.29 (0.25–0.33)	0.77 (0.09–2.76)	0.51 (0.32–0.77)
	70–74	NA	0.09 (0.05–0.15)	NA	0.14 (0.10–0.18)	NA	0.26 (0.19–0.34)	NA	0.49 (0.18–1.06)

Sensitivity (%) (95% CI)	50–59	95.8 (78.9–99.9)	93.7 (85.8–97.9)	89.4 (84.0–93.4)	81.2 (77.4–84.5)	84.2 (79.6–88.1)	70.3 (66.3–74.0)	76.2 (60.5–87.9)	52.9 (38.5–67.1)
	60–69	95.0 (75.1–99.9)	88.9 (84.2–92.6)	89.4 (80.8–95.0)	83.4 (81.0–85.7)	87.0 (76.7–93.9)	73.8 (70.5–77.0)	60.0 (14.7–94.7)	37.1 (21.5–55.1)
	70–74	NA	87.1 (79.9–92.4)	NA	87.4 (84.0–90.4)	NA	79.1 (73.3–84.1)	NA	45.5 (16.7–76.6)

Specificity (%) (95% CI)	50–59	93.8 (93.1–94.3)	98.5 (98.4–98.6)	89.6 (89.2–90.1)	97.4 (97.3–97.5)	89.3 (88.3–89.7)	96.9 (96.7–97.0)	92.4 (78.9–99.9)	97.2 (96.8–97.5)
	60–69	95.0 (93.9–95.9)	98.3 (98.2–98.4)	90.0 (89.1–90.9)	97.5 (97.4–97.6)	90.7 (89.6–91.7)	97.4 (97.3–97.5)	92.5 (88.6–95.4)	98.2 (97.7–98.6)
	70–74	NA	98.2 (98.0–98.4)	NA	97.5 (97.4–97.7)	NA	97.2 (97.0–97.4)	NA	97.7 (96.7–98.5)

Positive predictive value (%) (95% CI)	50–59	5.5 (3.5–8.1)	15.0 (12.0–18.5)	7.4 (6.4–8.6)	16.0 (14.6–17.5)	9.3 (8.3–10.5)	14.4 (13.1–15.8)	12.4 (8.6–17.1)	11.6 (7.8–16.4)
	60–69	17.3 (10.7–25.7)	21.2 (18.7–23.9)	14.2 (11.3–17.4)	23.3 (21.9–24.7)	17.6 (13.7–22.1)	24.1 (22.4–25.9)	13.6 (2.9–34.9)	14.3 (7.8–23.2)
	70–74	NA	25.1 (21.1–29.5)	NA	28.4 (26.1–30.9)	NA	26.1 (22.9–29.5)	NA	15.2 (5.1–31.9)

Negative predictive value (%) (95% CI)	50–59	100.0 (99.9–100.0)	100 (100.0–100.0)	99.9 (99.8–99.9)	99.9 (99.9–99.9)	99.8 (99.7–99.8)	99.8 (99.7–99.8)	99.6 (99.3–99.8)	99.7 (99.5–99.8)
	60–69	99.9 (99.7–100.0)	99.9 (99.9–100.0)	99.8 (99.8–99.9)	99.8 (99.8–99.9)	99.7 (99.4–99.8)	99.7 (99.4–99.8)	99.2 (97.0–99.9)	99.5 (99.2–99.7)
	70–74	NA	99.9 (99.8–99.9)	NA	99.9 (99.8–99.9)	NA	99.7 (99.6–99.8)	NA	99.5 (98.9–99.8)

**FIGURE A1:**
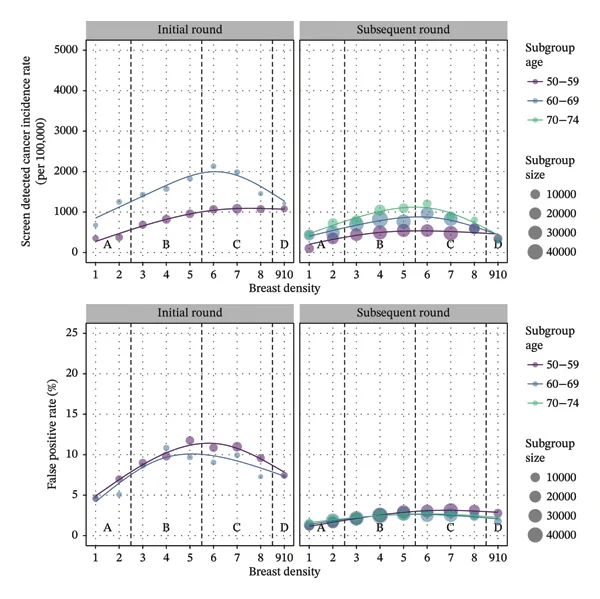
Proportion of screen‐detected cancers and false positive rates by age group, breast density and screening round.

**FIGURE A2:**
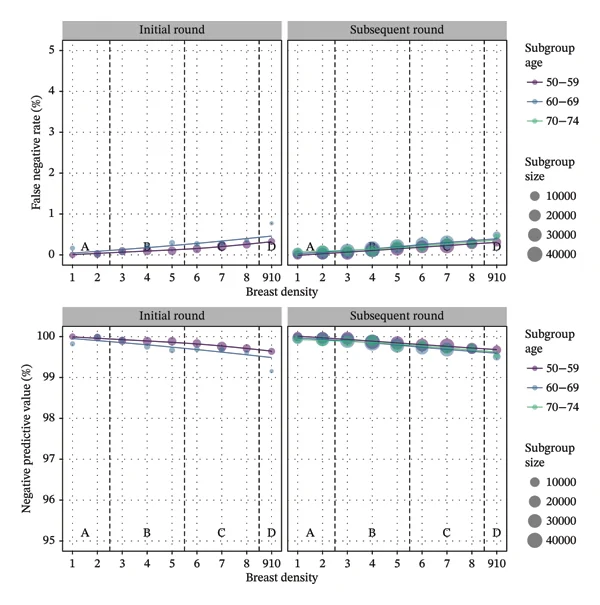
False negative rates and negative predictive value by age group, breast density and screening round.

The cancer incidence rate was higher for initial screeners at 1168 cases compared to 802 cases per 100,000 in subsequent round screeners, and also increased with age (709 in 50–59, 914 in 60–69 and 1041 in 70–74). The pattern in the incidence rate for screen‐detected cancers was similar to the overall cancer incidence, with the peak again observed in breast density C and lowest value in breast density A (Appendix Figures A1 and A2).

The relationship was noticeably different for interval cancers, with the proportion of interval cancers increasing with increasing breast density, even when accounting for a client’s age and screening round. Overall, 20.4% of cancers were interval cancers; this increased from 10.9% among clients with density A to 46.6% among those with density D.

**FIGURE 3:**
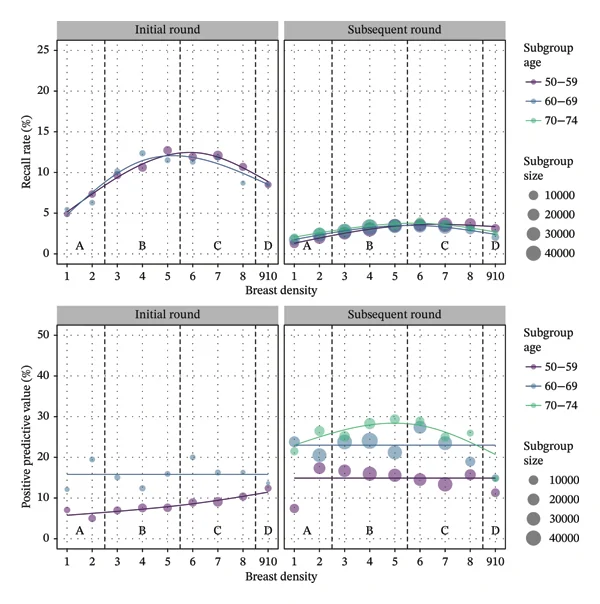
Recall rate and positive predictive value by age group, screening round and breast density (*x* axis, ordinal scale 1–10).

Recall rates (Figure 3), in general, were considerably higher for initial screeners (10.60%) compared to subsequent round screeners (3.08%). Recall rates followed an N‐shaped curve, similar to the pattern for screen‐diagnosed cancers, with the highest recall rates occurring among clients with breast density C. This pattern was the same for both initial and subsequent round screeners. Clients with density A had the lowest recall rates, while age had minimal impact on recall rates within screening rounds.

The relationship between positive predictive value (PPV) and breast density varied by screening round and age group. Among initial screeners aged 50–59 years, PPV increased with increasing breast density; however, no clear pattern was observed among initial screeners aged 60–69 years. Similarly, no consistent relationship between PPV and breast density was observed among subsequent round screeners. The highest PPV values occurred at different ordinal density scores across age groups (17.3% at density score 2 among clients aged 50–59 years, 27.1% at density score 6 among those aged 60–69 years and 29.5% at density score 4 among those aged 70–74 years). Across all density groups, PPV increased with increasing age.

**FIGURE 4:**
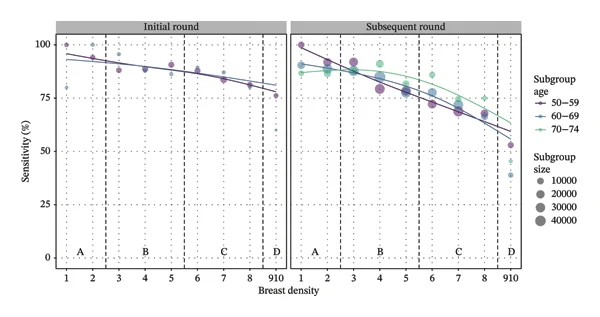
Program sensitivity by age group, screening round and breast density (*x* axis, ordinal scale 1–10).

Sensitivity (Figure 4) followed a consistent pattern across all age groups and screening rounds, with values declining as breast density increased. Among initial screeners, the profiles for clients aged 50–59 and 60–69 were very similar, although estimates for the 60–69 age group were more variable. This variability was most evident among the lowest and highest breast density scores, where the number of cancers was smaller. Among subsequent round screeners, sensitivity also declined with increasing breast density, with the reduction being more pronounced than among initial screeners. Sensitivity fell below 50% among clients with density D.

**FIGURE 5:**
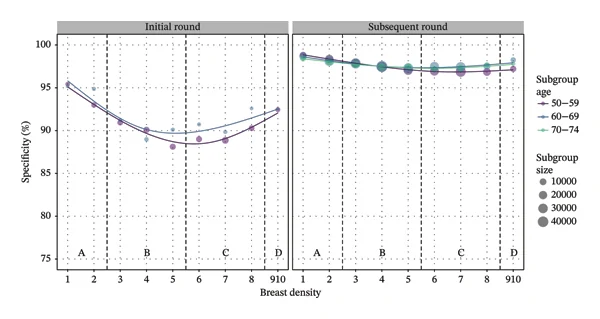
Program specificity by age group, screening round and breast density (*x* axis, ordinal scale 1–10).

Specificity (Figure 5) was lower for initial screeners compared with subsequent round screeners, with overall values of 90.3% and 97.5%, respectively. Across both screening rounds, the specificity curves followed a U‐shaped profile that mirrored the pattern observed for recall‐rate curves. This relationship is expected in the context of population‐based screening, where breast cancer remains relatively uncommon (approximately 843 cases per 100,000 people). The highest specificity values were observed among clients with breast density category A, while the lowest were seen among those with densities B and C. Specificity did not vary significantly with age.

## 4. Discussion

In this population‐based cohort study of BSNSW participants, we found that breast density was strongly associated with variation in screening performance. Clients with extremely dense breasts experienced lower screening sensitivity and, despite representing less than 3% of the screening population, accounted for a sizeable proportion of all interval cancers. In contrast, clients in the lowest breast density group experienced the lowest cancer incidence rate, the lowest rate of interval cancers and generally the lowest rates of recall, illustrating that the benefits and limitations of mammographic screening are not experienced uniformly across the screening population.

The findings of this study are broadly consistent with previous evidence demonstrating the importance of breast density as a determinant of mammographic screening performance. We found that sensitivity decreased with increasing breast density, a relationship that was independent of age and screening round. Specificity remained high throughout, particularly among clients in density class D (Figure 4), largely because recall rates were comparatively low (Figure 3). A plausible explanation may be the masking effect of dense tissue, which often appears homogeneous and opaque on mammography, concealing any underlying cancers. In the absence of a clear focal abnormality, radiologists are unable to recall clients, preserving high specificity but resulting in a marked reduction in sensitivity. These findings further suggest that intermediate levels of breast density may present the greatest interpretive challenge for screen readers, while the lack of variation in specificity by age indicates that age contributes little additional heterogeneity to this aspect of screening performance.

The study also highlighted that the relationship between cancer incidence rate and breast density is not linear and is complicated by the interaction with screening round and age. Importantly, it was found that cancer incidence and recall rates do not increase monotonically with breast density (when accounting for age and screening round), but instead follows an N‐shaped pattern with incidence rising through densities A to C but declining in density group D. This pattern may reflect a divergence between underlying cancer risk and screening detectability in women with extremely dense breasts. In a similar study, Payne et al. [20] have reported patterns in screening performance across breast density categories; however, the use of a large population‐based cohort and a more granular machine‐derived density measure has enabled a more detailed characterisation of these relationships here.

Additional observations included higher cancer incidence among initial screeners compared to subsequent round screeners; this difference likely reflects that first‐time screeners present with accumulation of breast cancer risk over a lifetime, compared with shorter interval risk between routine screens. Recall rates were lower and PPV were higher in subsequent round screeners. This may be because screen readers in the program have access to each client’s screening history, leading to increased confidence in assessments, particularly when no meaningful change is observed between screening episodes.

A major strength of this study is its large, consecutive, population‐based cohort, which captures the routine BSNSW screening population and minimises the potential for selection bias possible with smaller, study‐specific cohorts. The size of the cohort was particularly important given that interval cancers are relatively uncommon outcomes and may otherwise be estimated with limited precision. Furthermore, the application of machine‐reading technology enabled breast density to be assessed at scale using a more granular measure, allowing more detailed characterisation of the relationship between density and screening performance than is possible using conventional BI‐RADS categories alone. These findings also demonstrate the potential utility of machine‐derived breast density as an additional evidence source to inform future clinical and policy decision‐making.

This study also had several limitations. The imaging data from the screening episodes were collected approximately a decade ago; however, all images were acquired using digital mammography, which is still the standard modality used by BSNSW. In addition, the structure of the eligible screening population and cancer incidence rates are unlikely to have changed radically, meaning that the findings are still generalisable to a contemporary population‐based screening program. Interval cancers were pragmatically defined as any cancer diagnosed between screening episodes; however, some tumours may not have been visible at the time of screening or may have developed subsequently, meaning they could not have been detected. Finally, only unadjusted statistical comparisons are presented to avoid unnecessary complexity, meaning potential confounding factors were not accounted for in the analyses.

The limitations of screening mammography, particularly with regard to breast density, have been well documented [21, 22], yet addressing them in a large‐scale screening program has remained challenging. Priorities such as consistency of care, levels of service and cost efficiency have often taken precedence, deterring programs from pursuing a more individualised screening approach. However, the findings of this study demonstrate that screening performance varies substantially by breast density, highlighting inequities in the effectiveness of population‐based screening. By quantifying these differences, the findings contribute to the evidence base for future policy development by identifying the women most likely to benefit from alternative or supplementary screening approaches. In particular, the reduced sensitivity observed among women with extremely dense breasts raises the question of whether a uniform two‐dimensional mammography‐based approach is sufficient for all screening participants, or whether targeted use of alternative imaging modalities should be considered for those at the greatest risk of missed cancers. Future evaluation of approaches incorporating adjunct imaging modalities such as digital breast tomosynthesis (DBT), automated breast sonography (ABS), contrast‐enhanced mammography (CEM) or magnetic resonance imaging (MRI) will be required to determine their potential role in population‐based screening.

## 5. Conclusion

This study provides a population‐level assessment of the influence of breast density on the key performance metrics of a population‐based breast screening program. While the benefits of mammographic screening are well established, our findings demonstrate that screening performance varies considerably across breast density groups, with important implications for cancer detection and interval cancer risk. By quantifying these differences using a large representative cohort and a finer‐grain machine breast density assessment, this study provides evidence to support future research into risk‐stratified screening approaches and strategies to reduce inequities in screening performance.

## Author Contributions

CRediT

Chirag Mistry: methodology, software, investigation, visualisation, formal analysis and writing–original draft.

Bronwyn Morley: writing–review and editing.

Kan Ren: conceptualisation, methodology, writing–review and editing and validation.

David Roder: conceptualisation, methodology and writing–review and editing.

Richard Walton: conceptualisation, methodology, software, investigation, data curation, visualisation, formal analysis and supervision.

Matthew Warner‐Smith: conceptualisation, methodology, supervision and writing–review and editing.

Tracey O’Brien: writing–review and editing.

## Funding

This program of work is entirely conducted and supported by the Cancer Institute NSW, a pillar of NSW Health dedicated to state‐wide cancer control. No specific grant from any other institution or commercial entity has been received for this work.

## Conflicts of Interest

The authors declare no conflicts of interest.

## Data Availability

The data that support the findings of this study are available upon request from the corresponding author. The data are not publicly available due to privacy or ethical restrictions.
